# Licheniocin 50.2 and Bacteriocins from *Lactococcus lactis* subsp. *lactis* biovar. *diacetylactis* BGBU1-4 Inhibit Biofilms of Coagulase Negative Staphylococci and *Listeria monocytogenes* Clinical Isolates

**DOI:** 10.1371/journal.pone.0167995

**Published:** 2016-12-08

**Authors:** Ivana Cirkovic, Dragana D. Bozic, Veselin Draganic, Jelena Lozo, Tanja Beric, Milan Kojic, Biljana Arsic, Eliana Garalejic, Slobodanka Djukic, Slavisa Stankovic

**Affiliations:** 1 Institute of Microbiology and Immunology, Faculty of Medicine, University of Belgrade, Belgrade, Serbia; 2 Department of Microbiology and Immunology, Faculty of Pharmacy, University of Belgrade, Belgrade, Serbia; 3 Clinic for Gynecology and Obstetrics “Narodni front”, Faculty of Medicine, University of Belgrade, Belgrade, Serbia; 4 Faculty of Biology, University of Belgrade, Belgrade, Serbia; 5 Institute of Molecular Genetics and Genetic Engineering, University of Belgrade, Belgrade, Serbia; National University of Ireland - Galway, IRELAND

## Abstract

**Background:**

Coagulase negative staphylococci (CoNS) and *Listeria monocytogenes* have important roles in pathogenesis of various genital tract infections and fatal foetomaternal infections, respectively. The aim of our study was to investigate the inhibitory effects of two novel bacteriocins on biofilms of CoNS and *L*. *monocytogenes* genital isolates.

**Methods:**

The effects of licheniocin 50.2 from *Bacillus licheniformis* VPS50.2 and crude extract of bacteriocins produced by *Lactococcus lactis* subsp. *lactis* biovar. *diacetylactis* BGBU1-4 (BGBU1-4 crude extract) were evaluated on biofilm formation and formed biofilms of eight CoNS (four *S*. *epidermidis*, two *S*. *hominis*, one *S*. *lugdunensis* and one *S*. *haemolyticus*) and 12 *L*. *monocytogenes* genital isolates.

**Results:**

Licheniocin 50.2 and BGBU1-4 crude extract inhibited the growth of both CoNS and *L*. *monocytogenes* isolates, with MIC values in the range between 200–400 AU/ml for licheniocin 50.2 and 400–3200 AU/ml for BGBU1-4 crude extract. Subinhibitory concentrations (1/2 × and 1/4 × MIC) of licheniocin 50.2 inhibited biofilm formation by all CoNS isolates (p < 0.05, respectively), while BGBU1-4 crude extract inhibited biofilm formation by all *L*. *monocytogenes* isolates (p < 0.01 and p < 0.05, respectively). Both bacteriocins in concentrations of 100 AU/mL and 200 AU/mL reduced the amount of 24 h old CoNS and *L*. *monocytogenes* biofilms (p < 0.05, p < 0.01, p < 0.001).

**Conclusions:**

This study suggests that novel bacteriocins have potential to be used for genital application, to prevent biofilm formation and/or to eradicate formed biofilms, and consequently reduce genital and neonatal infections by CoNS and *L*. *monocytogenes*.

## Introduction

Coagulase negative staphylococci (CoNS) and *Listeria monocytogenes* are widely involved in minor to severe human infections, especially in immunocompromised patients. Despite their lower virulence, CoNS are well adapted to form strong biofilms and more recently, it has been noted that their biofilms have also an important role in pathogenesis of various genital tract infections, such as aerobic vaginitis and persistent nonspecific urethritis [1,2]. *L*. *monocytogenes* can colonise and live inside biofilms in the genital tract of women [3]. Although the incidence of *L*. *monocytogenes* in the vagina may be low [4], the consequences can be devastating, mostly in pregnancy. This bacterium could be transmitted to the neonate by ascending to the uterus or during passage of the foetus through the vagina and may cause significant morbidity and mortality in the neonates [3].

Biofilm is an assemblage of microorganisms embedded in an extracellular polymeric substance irreversibly attached to abiotic and/or biotic surfaces. Bacteria organized in multilayer structures differ in their phenotypic and genotypic features from their planktonic counterparts. Furthermore, biofilms also provide an ideal niche for the exchange of genes responsible for antimicrobial resistance [5,6]. According to present knowledge, more than 99% of bacteria in nature live in biofilms and in 80% of the human infections biofilms are shown to play an important role [7]. Therefore, biofilm-related infections are a therapeutic challenge of modern medicine.

Increasing antimicrobial resistance influenced enhanced interest in bacteriocins as alternative therapeutic means for treating infections. Bacteriocins are defined as antimicrobial peptides or proteins that usually act against closely related or non-related strains to bacteria that produced them [8,9].

Many recent studies have indicated possible practical applications of bacteriocins in food technology, but also in pharmacy and clinical medicine [10]. Interestingly, bacteriocins activity against *Neisseria gonorrhoeae* [11] and *Giardia lamblia* [12] was shown pointing to the bacteriocins as promising antimicrobial candidates for treatment of different pathogens. Furthermore, bacteriocins can inhibit growth of multiresistant pathogens of great importance, such as methicilin-resistant *Staphylococcus aureus* and vancomycin-resistant *Enterococcus* spp. [13]. The antibiofilm activity of various bacteriocins has recently been reported, but the greatest flaw of these investigations was that most of them were laboratory based studies, i.e. well-defined reference strains of microorganisms were used for testing [14], but not clinical isolates.

The aims of the present study were to investigate the effects of subinhibitory concentrations of bacteriocin licheniocin 50.2 and bacteriocins from *Lactococcus lactis* subsp. *lactis* biovar. *diacetylactis* BGBU1-4 on biofilm formation by clinical isolates of CoNS and *L*. *monocytogenes* and their antibiofilm activity on formed biofilms.

## Materials and Methods

### Purification and biochemical characterisation of bacteriocins

Purification and biochemical characterisation of bacteriocin licheniocin 50.2 from *Bacillus licheniformis* VPS50.2 was performed as previously described [15]. Crude extract of bacteriocins produced by *Lactococcus lactis* subsp. *lactis* biovar. *diacetylactis* BGBU1-4 (BGBU1-4 crude extract) was purified from cell-free supernatant of 16 h old overnight culture which was submitted to precipitation with ammonium sulfate to obtain 20, 30, 40, 50, and 60% of saturation. The resulting pellets were resuspended in 100 mM sodium phosphate buffer, pH 7 and tested for antimicrobial activity. The most potent fraction, 40% of saturation was submitted to further purification by chloroform extraction as follows. Equal volumes of resuspended pellet and chloroform were vigorously shaken for 15 min, left at +4°C for one hour and phases were divided by centrifugation at 13000rpm for 40 min. Obtained interphase was collected and dried in rotary vacuum evaporator (Eppendorf Concentrator 5301; Eppendorf). Dried interphase was resuspended in 100 mM sodium phosphate buffer, pH 7 and tested for bacteriocin activity against different indicator strains (*L*. *lactis* subsp. *lactis* BGMN1-596 [16], *L*. *lactis* subsp. *cremoris* NS1, *Lactobacillus paracasei* subsp. *paracasei* BGHN14 [17], *Lactobacillus plantarum* A112, *Listeria monocytogenes* ATCC 19111 and man-PTS deletion mutant of *L*. *lactis* subsp. *lactis* IL1403 (strain B464) [18]). Biochemical characterisation of BGBU1-4 and licheniocin 50.2 crude extracts was performed in order to determine pH range, influence of different temperatures (50°C, 60°C, 70°C, 80°C, 90°C and 100°C) and protelytic enzymes (protease, trypsin, chymotrypsin and pepsin) on bacteriocins activity.

### Bacterial isolates and growth conditions

In the present study, 8 strains of CoNS (4 strains of *Staphylococcus epidermidis*, 2 strains of *Staphylococcus hominis*, 1 strain of *Staphylococcus lugdunensis* and 1 strain of *Staphylococcus haemolyticus*) isolated from urethral swabs and 12 strains of *L*. *monocytogenes* isolated from vaginal swabs of patients from the Institute for Laboratory Diagnostics "Paster", Belgrade and Clinic for Gynaecology and Obstetrics “Narodni front”, Belgrade, were included in the further investigations. The study was approved by the Clinic for Gynaecology and Obstetrics “Narodni front” Ethical Committee [no. 24/22-2], and all patients signed informed consent form prior to their inclusion in the study.

Identification of the strains was performed in accordance with standard microbiology procedures and confirmed by automated Vitek2 System (bioMérieux, France). Clinical isolates were stored at -70°C in brain heart infusion broth (BHI; Lab M Limited, UK) with the addition of 10% sterile glycerol. Laboratory reference strains *S*. *epidermidis* ATCC 12228 and *L*. *monocytogenes* ATCC 19111 were used as positive controls. Prior to experiment, bacteria (clinical isolates and control strains) were defrosted, inoculated on tryptic soy agar (TSA; bioMérieux) and cultivated in aerobic conditions for 24 h at 35°C. Further, bacterial suspensions of grown cultures were prepared in sterile saline (bioMérieux) and adjusted to density of 0.5 McFarland standard (approximately 10^8^ CFU/ml) and then diluted to 10^6^ CFU/ml.

### Antimicrobial activity of bacteriocins

The antimicrobial activities spectrum of licheniocin 50.2 and BGBU1-4 crude extract were tested against clinical isolates and control strains of CoNS and *L*. *monocytogenes* with standard broth-microdilution test in accordance with Clinical Laboratory Standards Institute guidelines [19]. In short, serial dilutions of bacteriocins were prepared in fresh Mueller-Hinton broth (bioMérieux) with the addition of 0.05% triphenyl tetrazolium chloride (Sigma-Aldrich) as a growth indicator, set in triplicate and inoculated with 5x10^5^ CFU/mL of bacteria. Minimal inhibitory concentrations (MIC) were determined after incubation for 24 h at 35°C in aerobic conditions. Each test was repeated three times. The antimicrobial activity was expressed as arbitrary units (AU) per millilitre. AU is defined as reciprocal of the highest serial twofold dilution showing growth inhibition of the target strains [20].

### Effects of subinhibitory concentrations of licheniocin 50.2 and BGBU1-4 crude extract on biofilm formation by CoNS and *L*. *monocytogenes* strains

Biofilm formation was conducted in 96-well microtiter plates in accordance with Stepanović et al. [21]. Serial dilutions (1/2 × to 1/32 ×MIC) of licheniocin 50.2 and BGBU1-4 crude extract were prepared in fresh tryptic soy broth (TSB; bioMérieux) supplemented with 1% glucose (for CoNS) or brain heart infusion broth (BHI; Lab M Limited, UK) supplemented with 2% glucose and 2% saccharose (for *L*. *monocytogenes*). A 180 μL of each dilution was poured in triplicates into microtiter plate and aliquots (20 μL) of bacterial suspension were added to each well. Positive controls of each strain (bacteria in medium without presence of bacteriocins) were incubated under the same conditions. Negative controls for each plate were growth medium with or without bacteriocins. After 24 h of incubation at 35°C in aerobic conditions plates were decanted and rinsed gently three times with 300 μL of sterile phosphate-buffered saline (PBS; pH 7.2) to remove planktonic bacterial cells. After air drying plates were fixed with 150 μL methanol per well for 20 min, dried and stained with 150 μL (per well) of 2% crystal violet (bioMérieux) for 15 min. Unbound dye was rinsed with water. After air drying dye bound to the biofilm was released with 150 μL of 96% ethanol/well for 20 min. Optical density (OD) was measured at 570 nm using a microtiter plate reader (ICN Flow Titertek Multiscan Plus) following the calculation of the results according to Stepanović et al. [21]. Each assay was repeated three times. OD value of negative control (medium with bacteriocins) was subtracted from measured OD values of all tested strains and mean OD values from three experiments were calculated.

To calculate the category of biofilm formation, the cut-off optical density (ODc) was determined as three standard deviations above the mean OD of the negative control. According to the obtained results all tested strains were divided into four groups: OD ≤ ODc—category 0 (no biofilm producer); ODc < OD ≤ 2×ODc—category 1 (weak biofilm producer, +); 2×ODc < OD ≤ 4×ODc—category 2 (moderate biofilm producer, ++); 4×ODc < OD—category 3 (strong biofilm producer, +++).

### Effects of licheniocin 50.2 and BGBU1-4 crude extract on formed biofilm by CoNS and *L*. *monocytogenes*

To investigate the effects of licheniocin 50.2 and BGBU1-4 crude extract on formed biofilms, bacteria were cultivated in TSB or BHI medium in 96-well microtiter plates as previously described. After 24 h of incubation at 35°C in aerobic conditions plates were decanted and rinsed gently three times with 300 μL of sterile PBS. Subsequently, 24 h old biofilms were exposed to 100 AU/ml and 200 AU/ml of licheniocin 50.2 and BGBU1-4 crude extract (200 μl/well). Plates were incubated for additional 24 h at 35°C in aerobic conditions, rinsed, fixed and dyed as previously described, and the category of formed biofilm was calculated.

### Statistical analysis

The data obtained in this study were analysed in SPSS statistical program (PASW statistics for Windows, Version 18.0, Chicago: SPSS Inc. USA) using methods of descriptive statistics, Chi square test and Mann-Whitney U test.

## Results

### Biochemical characteristics and antimicrobial activity of bacteriocins

Biochemical characteristics of licheniocin 50.2 were previously described [15]. Both bacteriocins were active in a broad pH range (2–12), sensitive to different protelytic enzymes and heat stable.

The activities of licheniocin 50.2 and BGBU1-4 crude extract on planktonic cells of CoNS and *L*. *monocytogens* strains are presented in Table 1. Analysed bacteriocins inhibited the growth of both CoNS and *L*. *monocytogenes*, and the minimal inhibitory concentrations (MIC) were in the range between 200 and 400 AU/ml for licheniocin 50.2 and between 400 and 3200 AU/ml for BGBU1-4 crude extract. In addition, BGBU1-4 crude extract showed greater antimicrobial activity on *L*. *monocytogenes* strains than on CoNS.

**Table 1:** Minimal inhibitory concentrations of licheniocin 50.2 and BGBU1-4 crude extract and category of biofilm formation by coagulase negative staphylococci and *Listeria monocytogenes* clinical isolates.

Strain	Origin of the strain	MIC licheniocin 50.2 (AU/ml)	MIC BGBU1-4 crude extract (AU/ml)	Category of biofilm production	Mean OD±SD
*S*. *epidermidis*	urethral swab	200	200	2	0.25±0.04
*S*. *epidermidis*	urethral swab	200	200	3	0.62±0.04
*S*. *epidermidis*	urethral swab	200	200	2	0.23±0.06
*S*. *epidermidis*	urethral swab	200	200	3	0.62±0.07
*S*. *haemolyticus*	urethral swab	400	200	3	0.63±0.30
*S*. *hominis*	urethral swab	400	200	2	0.28±0.08
*S*. *hominis*	urethral swab	400	200	3	0.41±0.07
*S*. *lugdunensis*	urethral swab	400	200	2	0.20±0.02
*L*. *monocytogenes*	vaginal swab	400	3200	1	0.25±0.03
*L*. *monocytogenes*	vaginal swab	400	3200	3	1.03±0.09
*L*. *monocytogenes*	vaginal swab	400	3200	3	0.97±0.06
*L*. *monocytogenes*	vaginal swab	400	3200	2	0.55±0.02
*L*. *monocytogenes*	vaginal swab	400	3200	2	0.29±0.04
*L*. *monocytogenes*	vaginal swab	400	3200	2	0.36±0.01
*L*. *monocytogenes*	vaginal swab	400	3200	3	0.38±0.01
*L*. *monocytogenes*	vaginal swab	400	3200	3	1.13±0.07
*L*. *monocytogenes*	vaginal swab	400	3200	3	0.79±0.04
*L*. *monocytogenes*	vaginal swab	400	3200	3	0.59±0.03
*L*. *monocytogenes*	vaginal swab	400	3200	3	0.68±0.04
*L*. *monocytogenes*	vaginal swab	400	3200	3	1.11±0.08
*S*. *epidermidis* ATCC 12228	laboratory control strain	200	200	3	0.58±0.06
*L*. *monocytogenes* ATCC 19111	laboratory control strain	400	3200	2	0.25±0.02

MIC, minimal inhibitory concentration; AU, arbitrary units; OD, optical density; SD, standard deviation; 1, weak biofilm producer; 2, moderate biofilm producer; 3, strong biofilm producer.

### Biofilm formation by CoNS and *L*. *monocytogenes* strains

The evaluation of biofilm formation by CoNS and *L*. *monocytogenes* clinical isolates and control strains analysed in the study is presented in Table 1. All tested CoNS and *L*. *monocytogenes* clinical strains possess the ability to form biofilm: 4 (50%) strains of CoNS and 3 (25%) strains of *L*. *monocytogenes* were moderate biofilm producers, 4 (50%) strains of CoNS and 8 strains (66.7%) of *L*. *monocytogenes* were strong biofilm producers. Control strain *S*. *epidermidis* ATCC 12228 belongs to the category of strong and *L*. *monocytogenes* ATCC 19111 to the category of moderate biofilm producers. The presented OD values of analysed strains illustrated the individual variability of biofilm density between different isolates of bacteria belonging to the same species and same biofilm production category (Table 1).

### Effects of subinhibitory concentrations of licheniocin 50.2 and BGBU1-4 crude extract on biofilm formation by CoNS and *L*. *monocytogenes* strains

**Fig 1:**
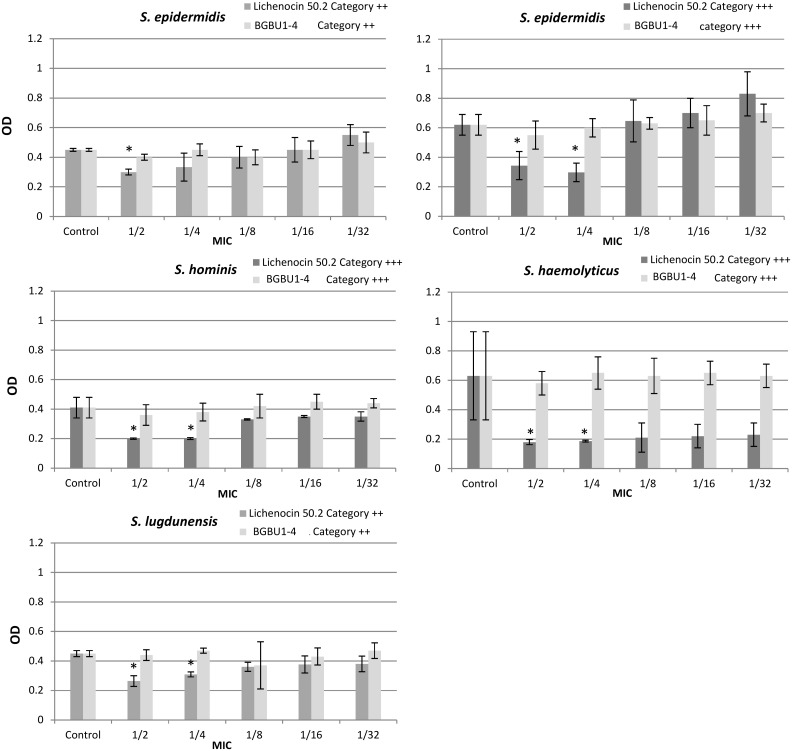
The effect of lichenocin 50.2 and BGBU1-4 crude extract on biofilm formation of coagulase negative staphylococci. The effect of subinhibitory concentrations (1/2–1/32 X MIC) of lichenocin 50.2 and BGBU1-4 crude extract on biofilm formation of *S*. *epidermidis*, *S*. *hominis*, *S*. *haemolyticus* and *S*. *lugdunensis*. Results are presented as mean OD ± standard deviation; Category ++, moderate biofilm producer; Category +++, strong biofilm producer; * p<0.05. Control–biofilm density of bacteria cultivated in medium without presence of bacteriocins.

**Fig 2:**
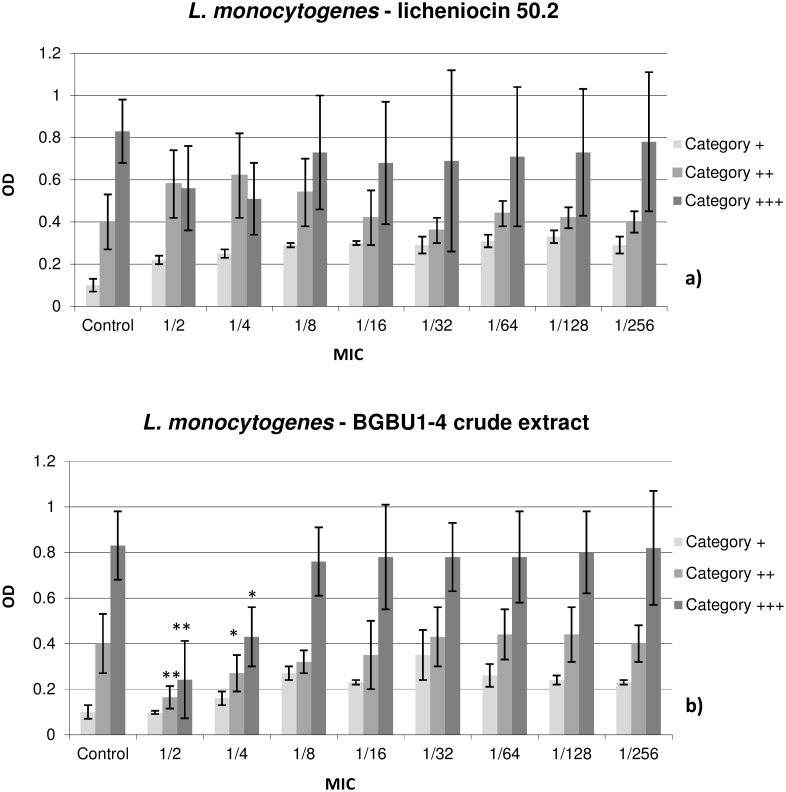
The effect of lichenocin 50.2 and BGBU1-4 crude extract on biofilm formation of *L*. *Monocytogenes*. The effect of subinhibitory concentrations (1/2–1/256 X MIC) of lichenocin 50.2 (2a) and BGBU1-4 crude extract (2b) on biofilm formation of *L*. *monocytogenes*. Results are presented as mean OD ± standard deviation; Category +, weak biofilm producer; Category ++, moderate biofilm producer; Category +++, strong biofilm producer; * p<0.05, ** p<0.01. Control–biofilm density of bacteria cultivated in medium without presence of bacteriocins.

The effects of subinhibitory concentrations of licheniocin 50.2 and BGBU1-4 crude extract on biofilm formation by CoNS and *L*. *monocytogenes* strains were examined in five concentrations (1/2 × to 1/32 × MIC) and presented in Figs 1 and 2. Significant activity of licheniocin 50.2 on biofilm formation by CoNS was obtained in 1/2 × and 1/4 × MIC concentrations (p < 0.05, respectively; Fig 1). All tested subinhibitory concentrations of licheniocin 50.2 had no significant inhibitory effects on biofilm formation by *L*. *monocytogenes* strains (p > 0.05; Fig 2a). Significant activity of BGBU1-4 crude extract on biofilm formation by *L*. *monocytogenes* was obtained in 1/2 × and 1/4 × MIC concentrations (p < 0.01, p < 0.05, respectively, Fig 2b). When concentration of 1/2 × MIC was used biofilm formation in 7.7% of *L*. *monocytogenes* strains (weak biofilm producers), 23.0% of strains (moderate biofilm producers) and 15.4% of strains (strong biofilm producers) was completely prevented. The effect of 1/4 × MIC dilution of BGBU1-4 crude extract was as following: 4 strains (30.8%) reduced biofilm production from moderate to weak, 3 strains (23.1%) from strong to weak and 2 strains (15.3%) from strong to moderate biofilm production (p < 0.05). Lower applied concentrations of BGBU1-4 crude extract had no effects on biofilm formation of *L*. *monocytogenes* and of CoNS strains compared to the positive control (p > 0.05; Figs 1 and 2).

### Effects of licheniocin 50.2 and BGBU1-4 crude extract on formed biofilm by CoNS and *L*. *monocytogenes* strains

The effects of both bacteriocins were tested in two concentrations (100 AU/mL and 200 AU/mL) on CoNS and *L*. *monocytogenes* formed biofilms. Both licheniocin 50.2 and bacteriocin BGBU1-4 crude extract were found to significantly contribute to eradication of 24 h old biofilms. Cultivation of 24 h old biofilm with 100 AU/mL and 200 AU/mL of licheniocin 50.2 significantly reduced the amount of formed biofilm by all tested strains of CoNS (p<0.01 and p<0.05, respectively). Similar effect was observed after cultivation of licheniocin 50.2 with formed biofilm of *L*. *monocytogenes* strains, with significant reduction of biofilm amount at concentrations of 100 AU/mL (p<0.001) and 200 AU/mL (p<0.01).

BGBU1-4 crude extract in concentration of 100 AU/mL and 200 AU/mL significantly reduced the amount of formed biofilm by all tested strains of CoNS (p<0.01, respectively) and all tested strains of *L*. *monocytogenes* (p<0.001 and p<0.01, respectively). In the presence of 100 AU/ml of BGBU1-4 crude extract, formed biofilm was completely eradicated in all (100%) *S*. *epidermidis* and *S*. *hominis* strains and in 76.9% of *L*. *monocytogenes* strains. Lower applied concentration of BGBU1-4 crude extract (200 AU/ml) completely eradicated formed biofilm in 50% of *S*. *epidermidis* and *S*. *hominis* strains and in 38.5% of *L*. *monocytogenes* strains. In both tested concentrations of BGBU1-4 crude extract, there were no significant differences (p>0.05) in eradication effects on formed biofilm between CoNS and *L*. *monocytogenes* strains.

## Discussion

In the last few decades, the exploration of new bacteriocins with enhanced biochemical features and broad antimicrobial activity has gained significance. In the present study, bacteriocin licheniocin 50.2 from *B*. *licheniformis* VPS50.2 and BGBU1-4 crude extract were investigated. Our previous work on these novel bacteriocins demonstrated that, due to their biochemical properties, they can be classified as class II bacteriocins. In order for it to be applied in different fields, it is important to be familiar with the mechanism of bacteriocins activity. So far five different target molecules involved in this have been identified [22]. Licheniocin 50.2 was not active against *Lactococcus lactis* ssp. *lactis* strain B464, derivative of IL1403 (man-PTS deletion mutant) suggesting that man-PTS is a receptor for activity of this bacteriocin [15]. Other class II bacteriocins, such as pediocin-like bacteriocin and lactococcin A employ man-PTS as a receptor [23,24]. However, preliminary tests showed that bacteriocin BGBU1-4 was active against B464, revealing that man-PTS is not a receptor for this bacteriocin (data not shown). It is known that class II bacteriocins have high bactericidal activities against many Gram-positive and Gram-negative bacteria, including CoNS and *L*. *monocytogenes* strains [14,25]. In the present study, the susceptibilities of the various staphylococcal species were diverse, especially to licheniocin 50.2. While *S*. *epidermidis* showed MIC values of 200 AU/ml for licheniocin 50.2, some other staphylococcal species, such as nonpathogenic *S*. *hominis*, *S*. *haemolyticus* or *S*. *lugdunensis*, were more susceptible. On the other hand, both bacteriocins demonstrate better antimicrobial activity on *L*. *monocytogenes* strains. It is well known that CoNS are more resistant to conventional antimicrobial agents compared to *L*. *monocytogenes* strains. However, in this study, CoNS were still sufficiently susceptible to consider licheniocin 50.2 and BGBU1-4 crude extract as a potential antimicrobial therapeutic agent.

At present, researches based on possible application of bacteriocins in medicine are of continuous interest. So far, there have been few reports on possible genital application of bacteriocins, for example effects of vaginal probiotics and their bacteriocins were evaluated on bacterial strains of non-genital origin [26], and so on. The vagina is subject to many disturbances which can result in vaginal pH value changes [27]. Bacterial vaginosis and aerobic vaginitis, two most common causes of abnormal vaginal discharge which are often mixed with other infections (bacterial infections, vulvovaginal candidiasis, *Trichomonas vaginalis*) are characterised by pH value ≥ 4,5 [28]. The administration of bacteriocins active in a broad pH range can be important for the treatment of vaginal infections and for maintaining a normal urogenital health. Furthermore, prior studies emphasised the possible role of CoNS on reproductive health of both men and women. CoNS are commonly isolated in patients with aerobic vaginitis and nonspecific urethritis [1,2]. Moreover, it has been shown that the contact of these bacteria with ejaculated spermatozoa can be a reason for severe injury of sperm membrane stability and mitochondrial activity with potential consequences on male fertility [29]. Additionally, *L*. *monocytogenes* can colonise genital tract and grow when there is an increase in pH [3] and in cases of pregnant women cause miscarriage, stillbirth, preterm labour and neonatal infections.

The main goal of this study was to evaluate activity of two novel bacteriocins against formed biofilms and biofilm formation. Biofilm-asocciated infections substantially affect human health, making them challenging to combat due to their antimicrobial resistance [5–7]. Hence, the prevention of biofilms is of utmost importance for modern medicine. The fact is that subinhibitory concentration of various antimicrobial agents possesses inhibitory effects on biofilm formation by different microorganisms [30,31]. On the other hand, it has also been reported that subinhibitory concentrations of some antimicrobial agents even enhance biofilm formation, for example erythromycin, nafcillin, quinupristin-dalfopristin, tetracycline and vancomycin on staphylococcal biofilm formation [32]. In our study, significant inhibition of biofilm formation was detected at 1/2 × and 1/4 × MIC for licheniocin 50.2 on CoNS and 1/2 × and 1/4 × MIC for BGBU1-4 crude extract on *L*. *monocytogenes*, suggesting that biofilm-relevant functions were affected at subinhibitory concentrations of both bacteriocins. This result indicated that licheniocin 50.2 and BGBU1-4 crude extract already exert an inhibiting effect on biofilm formation before they inhibit growth of CoNS and *L*. *monocytogenes*, respectively. Earlier studies showed that class I and class II bacteriocins inhibit transcription of genes involved in adherence and production of extracellular polymeric substances by staphylococci [32,33], implying it to be antibiofilm mechanism of licheniocin 50.2 and BGBU1-4 crude extract. Additionally, although licheniocin 50.2 showed similar activity on planktonic cells of CoNS and *L*. *monocytogenes*, subinhibitory concentration of this bacteriocin only affected staphylococcal biofilm formation. One hypothesis for this result could be the effect of dose or evidence of existence of different targets for bacteriocin action in biofilm formation process for various bacteria.

Once a biofilm has been established, the bacterial cells are extremely robust against all kinds of antimicrobial agents. Therefore, we studied the antibiofilm effects of licheniocin 50.2 and BGBU1-4 crude extract on 24 h old biofilms. Since the aim of the study was evaluation of possible genital application of these bacteriocins, two concentrations (100 AU/ml and 200 AU/ml) were analysed. Significant effects were seen with tested concentrations of licheniocin 50.2 and BGBU1-4 crude extract on formed biofilm of CoNS and *L*. *monocytogenes* strains. Moreover, these bacteriocins have an impact on formed biofilm of both bacteria, compared to the effects of subinhibitory concentration of bacteriocins where licheniocin 50.2 was active only on biofilm formation by CoNS and BGBU1-4 crude extract on biofilm formation by *L*. *monocytogenes*. Obtained results suggest that biofilm destruction by the bacteriocins was due not only to bacteria killing but also to destruction of the extracellular matrix and clearing of the biofilms from the surface.

## Conclusions

In conclusion, this study suggests that novel class II bacteriocins, licheniocin 50.2 and bacteriocins from BGBU1-4 crude extract, have potential to be used for genital application, to prevent biofilm formation and/or to eradicate biofilm formed by CoNS and *L*. *monocytogenes* strains in genital tract and consequently to reduce genital and neonatal infections. Further studies are in progress to evaluate the possible effects of these bacteriocins on other genital bacteria and their biofilms. Also, for future research, some mode of application and *in vivo* testing should take place.
